# Supplementary material for: Hepatitis B vaccination status and knowledge, attitude, and practice towards Hepatitis B virus among medical sciences students: A cross-sectional study
Source: PLoS One. 2023 Nov 6;18(11):e0293822. doi: 10.1371/journal.pone.0293822 (PMC10627443; doi:10.1371/journal.pone.0293822)
Supplement: Attachment — Submitted filename: PONE-D-23-22175_reviewer.docx [file pone.0293822.s002.docx]

PLOS ONE

**Hepatitis B Vaccination Status and Knowledge, Attitude, and Practice towards Hepatitis B virus among Medical Sciences Students: A Cross-Sectional Study**

--Manuscript Draft--

| **Manuscript Number:** | PONE-D-23-22175 |
| --- | --- |
| **Article Type:** | Research Article |
| **Full Title:** | Hepatitis B Vaccination Status and Knowledge, Attitude, and Practice towards Hepatitis B virus among Medical Sciences Students: A Cross-Sectional Study |
| **Short Title:** | Hepatitis B Vaccination Status and KAP towards Hepatitis B virus |
| **Corresponding Author:** | Ibrahim A Naqid, Ph.D University of Zakho  Zakho/Duhok, Kurdistan Region/Iraq IRAQ |
| **Keywords:** | Hepatitis B virus; vaccination; KAP; Medical Science students; Duhok; Iraq |
| **Abstract:** | Background and aims  Healthcare staffs are at high risk of occupational exposure to Hepatitis B and other blood-borne diseases. Lack of education about the knowledge of Hepatitis B virus contributes to the increase in cases. Our study aims to determine the knowledge of the Hepatitis B virus among the medical professionals in Duhok province, Kurdistan Region of Iraq, and to determine their knowledge of the importance of vaccination.  Materials and Methods  This cross-sectional study was conducted in the Duhok province of the Kurdistan region of Iraq among medical sciences students from November 2022 to February 2023. A Self-administered questionnaire comprising 22 items categorized into five sections was distributed to students either electronically or by paper and pen method. Microsoft Excel and GraphPad Prism 9 were used for statistical analysis.  Results  A total of 511 responses were collected from medical, dental, pharmacy, and laboratory students. The average age of the participants was (20.74 year +1.43). Among the respondents, only 96 (18.8%) were fully vaccinated against the Hepatitis B virus (received 3 or more doses of the vaccine), while 294 (57.5%) weren’t vaccinated. Lack of vaccination programs was the major reason for not receiving a vaccination (n=182, 62%). About 286 (55.96%) of the participants had good knowledge, attitude, and practice on Hepatitis B. The median knowledge, attitude, and practice score was 26, 18, and 20, respectively.  Conclusion  In our study, half of the students were found to be unvaccinated, mainly due to the absence of vaccination programs. Vaccinated students exhibited better knowledge, attitude, and practice toward the infection than non-vaccinated students. Therefore, we recommend the implementation of a vaccination program as well as training on infection prevention guidelines to increase awareness and encourage vaccination |
| **Order of Authors:** | Ibrahim A Naqid, Ph.D |
|  | Ahmed A Mosa |
|  | Shah Vahel Ibrahim |
|  | Nizar Hussein Ibrahim |
|  | Nawfal R Hussein |
| **Additional Information:** |  |

| **Question** | **Response** |
| --- | --- |
| **Financial Disclosure**  Enter a financial disclosure statement that describes the sources of funding for the work included in this submission. Review the [submission guidelines](http://journals.plos.org/plosone/s/submission-guidelines#loc-financial-disclosure-statement) for detailed requirements. View published research articles from [*PLOS ONE*](http://journals.plos.org/plosone/) for specific examples.  This statement is required for submission and **will appear in the published article** if the submission is accepted. Please make sure it is accurate. | The author(s) received no specific funding for this work. |
| **Unfunded studies**  Enter: *The author(s) received no specific funding for this work.*  **Funded studies**  Enter a statement with the following details:   - Initials of the authors who received each award - Grant numbers awarded to each author - The full name of each funder - URL of each funder website - Did the sponsors or funders play any role in the study design, data collection and analysis, decision to publish, or preparation of the manuscript? - **NO** - Include this sentence at the end of your statement: *The funders had no role in study design, data collection and analysis, decision to publish, or preparation of the manuscript.* - **YES** - Specify the role(s) played. |  |
| * typeset |  |
| **Competing Interests**  Use the instructions below to enter a competing interest statement for this submission. On behalf of all authors, disclose any [competing interests](http://journals.plos.org/plosone/s/competing-interests) that could be perceived to bias this work—acknowledging all financial support and any other relevant financial or non- financial competing interests. | The authors have declared that no competing interests exist. |

| This statement is required for submission and **will appear in the published article** if the submission is accepted. Please make sure it is accurate and that any funding sources listed in your Funding Information later in the submission form are also declared in your Financial Disclosure statement.  View published research articles from  [*PLOS ONE*](http://journals.plos.org/plosone/) for specific examples. |  |
| --- | --- |
| **NO authors have competing interests**  Enter: *The authors have declared that no competing interests exist.*  **Authors with competing interests**  Enter competing interest details beginning with this statement:  *I have read the journal's policy and the authors of this manuscript have the following competing interests: [insert competing interests here]* |  |
| * typeset |  |
| **Ethics Statement**  Enter an ethics statement for this submission. This statement is required if the study involved:   - Human participants - Human specimens or tissue - Vertebrate animals or cephalopods - Vertebrate embryos or tissues - Field research   Write "N/A" if the submission does not require an ethics statement.  General guidance is provided below. Consult the [submission guidelines](http://journals.plos.org/plosone/s/submission-guidelines#loc-guidelines-for-specific-study-types) for detailed instructions. **Make sure that all information entered here is included in the Methods section of the manuscript.** | The study protocol was approved by The Ethics and Scientific Committee in The College of Medicine, University of Zakho, Kurdistan Region of Iraq. All subjects provided Informed consent |

| **Format for specific study types**  **Human Subject Research (involving human participants and/or tissue)**   - Give the name of the institutional review board or ethics committee that approved the study - Include the approval number and/or a statement indicating approval of this research - Indicate the form of consent obtained (written/oral) or the reason that consent was not obtained (e.g. the data were analyzed anonymously)   **Animal Research (involving vertebrate animals, embryos or tissues)**   - Provide the name of the Institutional Animal Care and Use Committee (IACUC) or other relevant ethics board that reviewed the study protocol, and indicate whether they approved this research or granted a formal waiver of ethical approval - Include an approval number if one was obtained - If the study involved *non-human primates*, add *additional details* about animal welfare and steps taken to ameliorate suffering - If anesthesia, euthanasia, or any kind of animal sacrifice is part of the study, include briefly which substances and/or methods were applied   **Field Research**  Include the following details if this study involves the collection of plant, animal, or other materials from a natural setting:   - Field permit number - Name of the institution or relevant body that granted permission |  |
| --- | --- |
|  |  |
| **Data Availability**  Authors are required to make all data underlying the findings described fully available, without restriction, and from the time of publication. PLOS allows rare exceptions to address legal and ethical concerns. See the [PLOS Data Policy](http://journals.plos.org/plosone/s/data-availability) and [FAQ](http://journals.plos.org/plosone/s/data-availability#loc-faqs-for-data-policy) for detailed information. | Yes - all data are fully available without restriction |

| A Data Availability Statement describing where the data can be found is required at submission. Your answers to this question constitute the Data Availability Statement and **will be published in the article**, if accepted. |  |
| --- | --- |
| **Important:** Stating ‘data available on request from the author’ is not sufficient. If your data are only available upon request, select ‘No’ for the first question and explain your exceptional situation in the text box. |  |
| Do the authors confirm that all data underlying the findings described in their manuscript are fully available without restriction? |  |
| **Describe where the data may be found in full sentences. If you are copying our sample text, replace any instances of XXX with the appropriate details.**   - If the data are **held or will be held in a public repository**, include URLs, accession numbers or DOIs. If this information will only be available after acceptance, indicate this by ticking the box below. For example: *All XXX files are available from the XXX database (accession number(s) XXX, XXX.*). - If the data are all contained **within the manuscript and/or Supporting Information files**, enter the following: *All relevant data are within the manuscript and its Supporting Information files.* - If neither of these applies but you are able to provide **details of access elsewhere**, with or without limitations, please do so. For example:   *Data cannot be shared publicly because of [XXX]. Data are available from the XXX Institutional Data Access / Ethics Committee (contact via XXX) for researchers who meet the criteria for access to confidential data.*  *The data underlying the results presented in the study are available* | The data underlying the results presented in the study are available from (include the name of the third party and contact information or URL). |

| *from (include the name of the third party and contact information or URL).*   - This text is appropriate if the data are owned by a third party and authors do not have permission to share the data.   * typeset |  |
| --- | --- |
| Additional data availability information: |  |

[Click here to access/download;Manuscript;Manuscript KAP HB virus updated.docx](https://www.editorialmanager.com/pone/download.aspx?id=33662320&guid=88c3fd3a-5987-4595-82e8-e8e9a6b081bc&scheme=1)

**Hepatitis B Vaccination Status and Knowledge, Attitude, and Practice towards Hepatitis B virus among Medical Sciences Students: A Cross-Sectional Study**

# Ibrahim A. Naqid^1^, Ahmed A. Mosa^1^, Shah Vahel Ibrahim^1^, Nizar Hussein Ibrahim^1^ Nawfal R. Hussein^1^

**^1^Department of Biomedical sciences, College of Medicine, University of Zakho, Kurdistan Region, Iraq**

# Corresponding author:

Asst. Prof. Dr Ibrahim A. Naqid Department of Biomedical Sciences, College of Medicine, University of Zakho

Zakho International Road, Duhok, Kurdistan Region-Iraq

P.O. Box12

Email: [ibrahim.naqid@uoz.edu.krd](mailto:ibrahim.naqid@uoz.edu.krd) Tel: 009647504737593

# Abstract

**Background and aims:**

Healthcare staffs are at high risk of occupational exposure to Hepatitis B and other blood-borne diseases. Lack of education about the knowledge of Hepatitis B virus contributes to increase the cases. This study aims to determine the knowledge of the Hepatitis B virus among the medical professionals in Duhok province, Kurdistan Region of Iraq, and to determine their knowledge of the importance of vaccination.

**Materials and Methods:** This cross-sectional study was conducted in the Duhok province of the Kurdistan region of Iraq among medical sciences students from November 2022 to February 2023. A Self- administered questionnaire comprising 22 items categorized into five sections was distributed to students either electronically or by paper and pen method. Microsoft Excel and GraphPad Prism 9 were used for statistical analysis.

**Results:** A total of 511 responses were collected from medical, dental, pharmacy, and laboratory students. The average age of the participants was (20.74 ±1.43 year). Among the respondents, only 96 (18.8%) were fully vaccinated against the Hepatitis B virus (received 3 or more doses of the vaccine), while 294 (57.5%) were not vaccinated. Lack of vaccination programs was the major reason for not receiving a vaccination (n=182, 62%). About 286 (55.96%) of the participants had good knowledge, attitude, and practice on Hepatitis B. The median knowledge, attitude, and practice score was 26, 18, and 20, respectively.

**Conclusion:** In our study, half of the students were found to be unvaccinated, mainly due to the absence of vaccination programs. Vaccinated students exhibited better knowledge, attitude, and practice toward the infection than non-vaccinated students. Therefore, we recommend the implementation of a vaccination program as well as training on infection prevention guidelines to increase awareness and encourage vaccination

**Keywords:** Hepatitis B virus, Vaccination, KAP, Medical Science students, Duhok, Iraq

# Introduction

Hepatitis B virus (HBV) infection has been identified as the most common viral infection worldwide. Nearly two billion people have been infected with it, and nearly half a billion people are chronic carriers of the virus [1]. In the 2010 Global Burden of Disease study, the Hepatitis B virus was ranked as one of the tenth leading causes of death in the world, considering it as a major health issue and a priority [2]. The route of transmission of the Hepatitis B virus is through contact with infected blood or semen. There are three major modes of transmission. The first is the transmission from infected mothers to neonates prenatally. The second is the sexual transmission, especially among homosexuals, and people with positive records of sexually transmitted diseases. The third is due to unsafe injections, dialysis, and blood transfusions. Other possible routes of transmission include contaminated medical or surgical equipment, needle-stick injuries, and organs donated by HBs-Ag-positive or HBV-positive donors. Screening of blood donors for HBsAg has been a vital precaution in reducing the prevalence of the Hepatitis B virus [3].

Hepatitis B vaccines have been available in the United States since 1981 [4]. It is reported that with the increase of age, the antibody response declines. Immunization during childhood or adolescence offers the most potent protection against the virus. [5]. Infection with the Hepatitis B virus has been associated to predispose patients to develop liver diseases such as liver cirrhosis, liver failure, and hepatocellular carcinoma. The Hepatitis B vaccine has been effective in the prevention of hepatocellular carcinoma [6].

Healthcare staffs are at high risk of occupational exposure to Hepatitis B, Hepatitis C, and other blood-borne diseases. In developing countries, the poor emphasis on awareness and knowledge of blood-borne diseases puts the population at higher risk [7]. In 2017, a study in Duhok, Iraq focused on and interpreted the knowledge of Hepatitis B among healthy volunteers. In that study, it was reported that more than 40% of the participants did not know that the vaccine is available in Duhok city, and it was suggested that more awareness should be promoted in the region [8].

In 2016, a study was conducted in Duhok, Kurdistan Region, Iraq, to view the prevalence of Hepatitis B and C viruses among blood donors in the region [9]. According to the study, it was

stated that none of the donors obtained vaccination against HBV due to initiation of vaccination programs began in 2003, which meant that only people younger than 12 years old were vaccinated. Secondly, the unavailability or high cost of the vaccine was a serious barrier for not inoculated in the region. Lastly, the lack of awareness of the vaccine had a huge role in poor vaccination status among the donors. Lack of education about the knowledge of Hepatitis B virus aids in increasing the disease. There are high-risk factors in the Duhok region as well, including drug users sharing contaminated needles, sexual encounters with infected people, and healthcare staff [10,11].

# Aims of the study

Our study aims to determine the knowledge, attitude and practice towards Hepatitis B virus among the medical sciences in Duhok, Iraq, and to determine their knowledge on the importance of vaccination. This will help in encouraging awareness and educating medical sciences’ staff, and the population in general, to protect themselves from the virus, and advocate for the effective role of the vaccine against the disease initiation and progression.

# Materials and Methods Study Design

This cross-sectional study was carried out among medical sciences students in the Duhok province, Kurdistan region of Iraq. The data were collected from November 2022 to February 2023, and a total of 511 students were recruited for the study. A self-administered questionnaire was delivered to students either electronically using the Google forms platform or via the paper and pen method. The participant's anonymity was protected and participation was entirely voluntary.

# Study Tool

The study survey was derived from a previously validated questionnaire , with certain adjustments made by the authors to meet the current study aims [12]. The survey questionnaire comprised 22 items which were categorized into five sections. The first section included three questions related to the basic demographic characteristics of participants like age, gender, and college. The second section featured three items regarding the vaccination status, Hepatitis B vaccine doses, and the reason for not being vaccinated.

The study’s last part comprised questions designed to examine students’ knowledge, attitude, and practice on Hepatitis B, which may be useful in understanding students’ acceptance and reticence regarding the hepatitis B vaccine. This part of the questionnaire was assorted into three sections: six items to test participants’ hepatitis B knowledge, five items to assess participants’ attitudes regarding the virus, and the final five questions to address students’ practice.

Responses to the questions about knowledge, attitude, and practice were measured using a Five- point Likert scale, ranging from 1-5 from strongly disagree to strongly agree., However, one question in the knowledge section (Hepatitis B can be transmitted by shaking hands, coughing/sneezing, and contaminated food/water) and two questions in the attitude section (I feel uncomfortable sitting with a hepatitis B infected person) and (I don't need hepatitis B vaccination because I'm not at risk) were reversed scored.

Knowledge of hepatitis B was examined adding the six questions with a potential score ranging from 6-30, while Attitude and Practice sections were assessed adding five questions with a possible score ranging from 5-25.

# Inclusion/Exclusion criteria

The inclusion criteria were participants of more than 18 years of age, students in one of the medical sciences colleges in Duhok province, and consenting to be recruited in the study. While non-medical sciences students, students from other provinces, and data with incomplete information were excluded from the study.

# Ethical approval

The study protocol was approved by The Ethics and Scientific Committee of The College of Medicine, University of Zakho, Kurdistan Region of Iraq. All subjects provided Informed consent

# Statistical analysis

Statistical analysis was performed using Microsoft Excel and GraphPad Prism 9 software. Frequencies and percentages were used to describe descriptive statistics of the participants. The association between basic-demographic characteristics variables and KAP score was studied by using Chi-Square, or Fisher's exact test. Statistical significance was defined as a p-value of 0.05 or less.

# Results

**Basic demographic characteristics**

The basic demographic characteristics of the respondents who participated in this study are shown in **Table 1**. The mean age of the participants was 20.74 (±1.43 SD) years. Two hundred seventy one respondents were female (53%).. The majority of the respondents were medical students (n=248, 48.5%) followed by dental students (n=161, 31.5%). Regarding the vaccination status, 217 (42.5%) of the students were vaccinated while 294 (57.5%) were not vaccinated. Among the vaccinated students, 96 (44.2%) students were fully vaccinated (received 3 or more doses of the vaccine). The lack of a vaccination program was the major reason for not being vaccinated (n=182, 62%).

**Table 1. Demographic characteristics of the participants**

| **Variables** | **n (%)** |
| --- | --- |
| **Age, Mean (SD)** | 20.74 (1.43) |
| **Gender** |  |
| Male | 240 (47) |
| Female | 271 (53) |
| **Field of study** |  |
| College of Medicine | 248 (48.5) |
| College of Dentistry | 161 (31.5) |
| College of Pharmacy | 52 (10.2) |
| Medical Laboratory College | 50 (9.8) |
| **Vaccinated against hepatitis B** |  |
| Yes | 217 (42.5) |
| No | 294 (57.5) |
| **Doses of hepatitis B vaccine received** |  |
| Not vaccinated | 294 (57.5) |
| One | 41 (8) |
| Two | 80 (15.7) |
| Three | 87 (17) |
| More than three | 9 (1.8) |
| **Reasons for not being vaccinated against Hepatitis B** |  |
| No vaccination program offered | 182 (62) |
| Low risk of hepatitis B | 26 (8.8) |
| Not sure about the vaccination status | 22 (7.5) |
| Lack of knowledge | 36 (12.2) |
| Efficacy doubted | 28 (9.5) |

# Assessment of knowledge related to Hepatitis B virus

**Table 2** shows the knowledge of the participants on Hepatitis B. Most of the respondents agreed that Hepatitis B is caused by a virus (67.5% strongly agreed, 24.1% agreed) and can cause liver cancer (35.6% strongly agreed, 38.6% agreed). Regarding the knowledge of the participants on the mode of transmission, the majority of the students agreed that Hepatitis B can be transmitted by contaminated blood, body fluids, and unprotected sex (62.2% strongly agreed, 25.4% agreed). Similarly, most of the students disagreed that Hepatitis B can be transmitted by shaking hands, coughing/sneezing, and contaminated food/water (48.7% strongly disagreed, 27.2% disagreed) and 11% were neutral. About four-fifths of the participants agreed that healthcare workers are at increased risk of contracting Hepatitis B than the general population (50.9% strongly agreed, 28.2% agreed). In terms of knowledge on the prevention, the majority of the students agreed that Hepatitis B can be prevented by vaccination, using gloves, and avoiding sharp needles/syringe injury (55.8% strongly agreed, 34.2% agreed).

# Table 2. Assessment of Knowledge related to Hepatitis B virus

| **Variables** | Strongly  Disagree n (%) | Disagree n (%) | Neutral n (%) | Agree n (%) | Strongly Agree n (%) |
| --- | --- | --- | --- | --- | --- |
| Hepatitis B is caused by a virus. | 10 (2) | 8 (1.6) | 25 (4.9) | 123 (24.1) | 345 (67.5) |
| Hepatitis B can be transmitted by contaminated blood, body fluids, and unprotected sex. | 11 (2.2) | 22 (4.3) | 30 (5.9) | 130 (25.4) | 318 (62.2) |
| Hepatitis B can be transmitted by shaking hands, Coughing/Sneezing, and contaminated food/water. | 249 (48.7) | 139  (27.2) | 56 (11) | 38 (7.4) | 29 (5.7) |
| Hepatitis B can cause liver cancer. | 13 (2.5) | 23 (4.5) | 96 (18.8) | 197 (38.6) | 182 (35.6) |
| Healthcare workers are at increased risk of getting Hepatitis B than general population. | 14 (2.7) | 26 (5.1) | 67 (13.1) | 144 (28.2) | 260 (50.9) |
| Hepatitis B can be prevented by vaccination, using gloves and avoiding sharp needles/syringe injury. | 9 (1.8) | 10 (2) | 32 (6.3) | 175 (34.2) | 285 (55.8) |

**Assessment of attitude towards Hepatitis B virus**

**Table 3** shows the attitude of the participants toward Hepatitis B. Many of the students were neutral (n=140, 27.4%) toward sitting with a Hepatitis B infected person, however, 14.3% (n=73) of the participants strongly agreed on feeling uncomfortable sitting with a Hepatitis B infected person and 14.5% (n=74) strongly disagreed. Twenty seven percent (n=138) disagreed on shaking hands/hugging with an infected person, while 21.3% (n=109) don’t mind shaking hands/hugging with a hepatitis B infected person and 25.4% (n=130) were neutral. About 41.1% (n=210) of students agreed that the Hepatitis B vaccine is safe and effective and 3.9% (n=20) strongly disagreed. About two-thirds of the participants (66.7%, n=341) strongly agreed that healthcare workers should receive Hepatitis B vaccination. About half of the respondents (52.6%, n=269) strongly disagreed with not receiving Hepatitis B vaccination because they are not at risk.

# Table 3. Assessment of Attitude towards Hepatitis B virus

| **Variables** | Strongly  Disagree n (%) | Disagree  n (%) | Neutral  n (%) | Agree n  (%) | Strongly  Agree n (%) |
| --- | --- | --- | --- | --- | --- |
| I feel uncomfortable sitting with a hepatitis B  infected person. | 74 (14.5) | 93 (18.2) | 140  (27.4) | 131  (25.6) | 73 (14.3) |
| I don't mind shaking hands/hugging with a  hepatitis B infected person. | 77 (15.1) | 138 (27) | 130  (25.4) | 109  (21.3) | 57 (11.2) |
| I believe the hepatitis B vaccine is safe and  effective. | 20 (3.9) | 19 (3.7) | 76 (14.9) | 210  (41.1) | 186 (36.4) |
| I believe healthcare workers should receive  hepatitis B vaccination. | 6 (1.2) | 16 (3.1) | 37 (7.2) | 111  (21.7) | 341 (66.7) |
| I don't need hepatitis B vaccination because I'm  not at risk. | 269 (52.6) | 142  (27.8) | 45 (8.8) | 30 (5.9) | 25 (4.9) |

**Assessment of practice towards Hepatitis B virus**

**Table 4** shows the practice of the respondents toward Hepatitis B.

Among the participants, 38.2% (n=195) ask/use a new blade for shaving/hair cutting. 60.5% (n=319) of the respondents ask for a new syringe before injection. About half of the students (50.3%, n=257) ask for sterilized equipment for ear/nose piercing. 42.5% (n=217) will report for needle prick/ sharp injuries. 27.6% (n=141) were neutral on attending Hepatitis B awareness

campaigns and 13.9% (n=71) strongly disagreed.

# Table 4. Assessment of Practice towards Hepatitis B virus

| **Variables** | Strongly Disagree n (%) | Disagree n (%) | Neutral n (%) | Agree n (%) | Strongly Agree n (%) |
| --- | --- | --- | --- | --- | --- |
| I ask/use a new blade for shaving/hair cutting. | 26 (5.1) | 43 (8.4) | 103  (20.2) | 144  (28.2) | 195 (38.2) |
| I ask for a new syringe before injection. | 8 (1.6) | 23 (4.5) | 48 (9.4) | 123  (24.1) | 309 (60.5) |
| I ask for sterilized equipment for ear/nose piercing. | 8 (1.6) | 24 (4.7) | 87 (17) | 135  (26.4) | 257 (50.3) |
| I will report for needle prick/ sharp  injuries. | 4 (0.8) | 32 (6.3) | 107  (20.9) | 151  (29.5) | 217 (42.5) |
| I attend hepatitis B related awareness campaigns. | 71 (13.9) | 68 (13.3) | 141  (27.6) | 139  (27.2) | 92 (18) |

**Categorization of knowledge, attitude, and practice (KAP) score and its association with basic demographic characteristics**

The total median score of KAP was 65 as shown in **Table 5**. The median knowledge, attitude and practice score was 26,19,20, respectively **(Table 5)**. As shown in **Figure 1**, 51.5% of the study participants had a favorable KAP score (above the median score). **Table 6** shows the association between KAP score and basic demographic characteristics including gender, field of study (college), and vaccination status. We found a significant association between KAP score and the field of study (p=0.0002) and vaccination status (p=0.0003). However, there was no significant association between gender (0.093) and KAP score **(Table 6).**

# Table 5. Summation of Knowledge, attitude, and practice score distribution

|  | **Knowledge sum**  **(n=511)** | **Attitude sum**  **(n=511)** | **Practice sum**  **(n=511)** | **Total Score (n=511)** |
| --- | --- | --- | --- | --- |
| **Mean** | 25.61 | 18.5 | 19.7 | 63.81 |
| **Median** | 26 | 19 | 20 | 65 |

**Figure 1. Participants group based on KAP score. Good score ≥65; Inadequate** <**65**

**Inadequate**

**248**

**Good**

**263**

**0**

**50**

**100**

**150**

**200**

**250**

**300**

**350**

**Student Number**

Participants based on KAP Score

# Table 6. Association of KAP score with basic-demographic characteristics

| **Variables** | | **KAP score Category** | | ***p-value** |
| --- | --- | --- | --- | --- |
|  |  | In-adequate (<65) | Good (≥65) |  |
| **Gender** | Male | 126 | 114 | 0.093 |
|  | Female | 122 | 149 |  |
| **Field of study (College)** | College of Medicine | 112 | 136 | 0.0002 |
|  | College of Dentistry | 73 | 88 |  |
|  | College of Pharmacy | 22 | 30 |  |
|  | Medical Laboratory College | 41 | 9 |  |
| **Vaccinated against Hepatitis B** | Yes | 85 | 132 | 0.0003 |
|  | No | 163 | 131 |  |

***P value is measured using Chi-Square, or Fisher's exact test.**

# Discussion

Medical sciences students are at high risk of exposure to blood-borne infections such as Hepatitis B virus, as they provide direct patient care throughout the clerkship phase of the program, this puts them at a comparable to a higher risk than other health care professionals. Therefore, it is imperative that all students should be vaccinated and acquire sufficient knowledge about the virus to minimize the risk of infection. In the Kurdistan region of Iraq, there have been no studies to measure vaccination status as well as knowledge, attitude, and practice towards the Hepatitis B virus among medical sciences students, as far as authors’ knowledge are concerned. This study is designed to evaluate the vaccination status of medical sciences students’ and their KAP about the virus.

In our study, 42.5% of medical science students were vaccinated against Hepatitis B, which is higher than the findings of a study in Southwest Ethiopia, where only 25.7% of students were immunized [13]. But our results are similar to a study from Pakistan, where 42.2% of students were vaccinated, and are quite lower than a study from Nigeria, where 47.7% of students reported that they have been vaccinated [14,15]. Also, it is lower than a research carried out in Uganda, where it was found that 66.8% of the participants had received vaccinations [16]. In all, 18.8% of participants were fully vaccinated (3 doses or more), which is much greater than a study in Ethiopia, where just 2% of students were fully vaccinated [17]. In comparison to our findings, a research in Nepal found that 37% of their students had completed all three doses of vaccination, which is greater than our study [12].

The primary reasons for non-being vaccinated among 57.5% of our study’s unvaccinated students, were no vaccination program offered 62% followed by lack of knowledge 12.2% and efficacy doubted 9.5%. Our results are in line with other studies conducted in Nepal and Nigeria, which found a lack of effective vaccination programs 43.2% and lack of opportunity 57.4% as the main reason for non-vaccination, respectively. [15,18]. These findings highlight the need for urgent implementation of vaccination programs for medical science students in the region. In Uganda, the high cost of vaccination 63.2% was the most common obstacle for non-vaccination [16].

About 91.6% of the participants surveyed were aware that Hepatitis B is caused by a virus. Comparing to research carried out in Nepal among medical and dental students showed that 93.6% of the students knew that Hepatitis B infection was caused by a virus [19]. The great majority of participants have a solid understanding of the mode of transmission, with 87.6% stating that transmission occurs through contaminated blood, fluid, and unprotected intercourse. These findings are consistent with those of Ethiopian and Nepalese studies [12,17]. In the present study, 74.2% of participants agreed that the Hepatitis B virus can lead to liver cancer. This is comparable with researches from Saudi Arabia, Nepal, and Ethiopia which revealed that 75.5%, 80.6%, and 81.3% of students agreed that Hepatitis B infection can cause liver cancer, respectively [12,17,20]. 90% of respondents agreed that vaccination may prevent Hepatitis B infection, comparable results were observed in studies carried out in Saudi Arabia, Nepal, and Ethiopia [12,17,20].

Around 39.9% and 42.1% of students showed a negative attitude towards sitting and shaking hands with a Hepatitis B infected person, respectively. Nepalese students demonstrated a more positive attitude [12]. In the present study, 77.5% of the students believed that the Hepatitis B vaccine was safe and efficient; somewhat higher results were obtained from studies conducted in Saudi Arabia, Nepal, and Ethiopia [12,17,20]. While, compared to another study in Saudi Arabia our findings are higher where only 63% of participants agreed that vaccine is safe and effective [21]. 88.4% of our students agreed that healthcare workers should be vaccinated, which is similar to a study from Nepal [12]. According to our survey, 10.8% of participants believed that they are not at risk of contracting Hepatitis B virus and that vaccination against the Hepatitis B is not necessary; this figure nearly triples when compared to studies conducted in India and Nepal, whereas only 3.7% and 3.9% of their participants agreed to this statement, respectively [12,22]. This concerning finding should be addressed because medical sciences’ students are part of the healthcare system and should be aware of the need of Hepatitis B vaccination since they are vulnerable of contracting the virus and spreading the infection in the community.

In our survey, 66.4%, 84.6%, and 76.7% of the students stated that they requested a new blade for shaving/hair cutting, new syringes to avoid infection, and sanitized equipment for piercing their ears and nose. Studies from India and Nepal reported better safety practices among their

participants [12,22]. These findings highlight the necessity of taking action that encourages students to adhere to infection control measures. 72% of the students agreed that they will report needle prick/sharp injuries, our finding is greater than the findings of Saudi Arabian, Nepalese, and Ethiopian studies which found that 68%, 64.6%, and 53.7% of their participants will report needle injuries, respectively [12,17,20]. 45.2% of the study students said that they have attended Hepatitis B related awareness programs which is similar to the findings of a study in Nepal 44.2% [12].

In this study, we found that the female gender is associated with better overall KAP scores when compared to males, similarly reported in the previous studies from Pakistan and Nepal [12,23]. However, a study from Malaysia revealed no association between gender and knowledge about Hepatitis B [24]. Students who were vaccinated showed a better KAP score than those who weren’t, a similar result was reported by a Nepalese study [12].

# Limitations and Strengths of the study

The main limitation of this research is that vaccination status was self-reported and not confirmed by the measurement of the anti-Hepatitis B surface antibody (HBsAb) titer of the students. Therefore, recall bias, and erroneous information might have affected the findings of the research. Since the study was conducted only in Duhok province in the Kurdistan Region of Iraq the results cannot be generalized to all Medical sciences Colleges in the region, but they will undoubtedly serve as a background for future studies in the region. Finally, the nature of the study was cross-sectional, and it didn’t measure the cause-and-effect relationship. Despite the limitations, the study was strengthened by the large sample size used to measure individuals’ vaccination status and KAP against Hepatitis B virus, which minimized the likelihood of bias.

# Conclusion

In the present study, more than half of the participants were not vaccinated against Hepatitis B despite it being a preventable disease, and only one-fifth of students were fully vaccinated. The lack of vaccination programs was the main reason for not being vaccinated against Hepatitis B among the study participants. Moreover, about half of the participants had good knowledge,

attitude, and practice (KAP) regarding Hepatitis B, and students who were vaccinated displayed a better KAP score than those who were non-vaccinated. The administrators of the medical sciences college should prioritize the implementation of a vaccination program.

# Recommendations

Since all medical sciences students are susceptible to the Hepatitis B infection as they are exposed to the infected blood and blood products in their professional career. Therefore, all students should be vaccinated against the Hepatitis B virus upon entry to the college. The COVID- 19 pandemic has had a significant impact on the region’s healthcare system including medical education [25-27]. This had negatively impacted and postponed many infection prevention programs in the Kurdistan Region [28]. This might be linked to low vaccination coverage in our study. Therefore, we recommend the implementation of a vaccination program and provision of training on infection prevention guidelines to increase students’ knowledge, attitude, and practice toward Hepatitis B.

**Funding:** This research received no external funding

**Conflicts of Interest:** The authors declare no conflict of interest

**Acknowledgments:** We would like to express sincere gratitude to all students who took part in this study.

# Author contributions

Study concept and design: **A.M, S.I** and **N.I.**; analysis and interpretation of data: **A. M.**, and **I.N;** drafting of the manuscript: **A.M, S.I** and **N.I**; critical revision of the manuscript for important intellectual content: **I.N and N.H.**; statistical analysis: **A.M.** All authors read and approved the final manuscript

**Informed Consent:** Informed consent was obtained from all subjects involved in the study

**Data Availability:** The datasets used in this work are available upon request from the corresponding author.

**References**

1. Liaw YF, Chu CM (2009) Hepatitis B virus infection. Lancet 373: 582-592.
2. Lozano R, Naghavi M, Foreman K, Lim S, Shibuya K, et al. (2012) Global and regional mortality from 235 causes of death for 20 age groups in 1990 and 2010: a systematic analysis for the Global Burden of Disease Study 2010. Lancet 380: 2095-2128.
3. Trépo C, Chan HL, Lok A (2014) Hepatitis B virus infection. Lancet 384: 2053-2063.
4. Poland GA, Jacobson RM (2004) Clinical practice: prevention of hepatitis B with the hepatitis B vaccine. N Engl J Med 351: 2832-2838.
5. Fisman DN, Agrawal D, Leder K (2002) The effect of age on immunologic response to recombinant hepatitis B vaccine: a meta-analysis. Clin Infect Dis 35: 1368-1375.
6. Lu T, Seto WK, Zhu RX, Lai CL, Yuen MF (2013) Prevention of hepatocellular carcinoma in chronic viral hepatitis B and C infection. World J Gastroenterol 19: 8887-8894.
7. Singhal V, Bora D, Singh S (2009) Hepatitis B in health care workers: Indian scenario. J Lab Physicians 1: 41-48.
8. Abdullah RA, Badal R, Hussein NR, Jaafar S, Yaqoob ES, et al. (2017) Hepatitis B Knowledge Among Healthy Volunteers in Duhok City, Kurdistan Region, Iraq. 4: e14976.
9. Rasheed Hussein N, Mohamad Haj S, Amin Almizori L, Ahmed Taha A (2017) The Prevalence of Hepatitis B and C Viruses Among Blood Donors Attending Blood Bank in Duhok, Kurdistan Region, Iraq. 4: e39008.
10. Khalid FK, Rasheed NA, Hussein NR, Naqid IA (2022) A study of HBV infection and its risk factors in pregnant women in Zakho city, Iraq. PLOS ONE 17: e0273362.
11. Hussein NR, Musa DH, Hawezy DJ, Ahmed FMR, Khalid FK, et al. (2021) A study on the prevalence and the risk factors of hepatitis B virus infection in Kurdistan Region, Iraq: A multicenter study: Prevalence and the risk factors of HBV. Journal of Contemporary Medical Sciences 7.
12. Shrestha DB, Khadka M, Khadka M, Subedi P, Pokharel S, et al. (2020) Hepatitis B vaccination status and knowledge, attitude, and practice regarding Hepatitis B among preclinical medical students of a medical college in Nepal. PLoS One 15: e0242658.
13. Haile K, Timerga A, Mose A, Mekonnen Z (2021) Hepatitis B vaccination status and associated factors among students of medicine and health sciences in Wolkite University, Southwest Ethiopia: A cross-sectional study. PLOS ONE 16: e0257621.
14. Nasir K, Khan KA, Kadri WM, Salim S, Tufail K, et al. (2000) Hepatitis B vaccination among health care workers and students of a medical college. J Pak Med Assoc 50: 239-243.
15. Okeke EN, Ladep NG, Agaba EI, Malu AO (2008) Hepatitis B vaccination status and needle stick injuries among medical students in a Nigerian university. Niger J Med 17: 330-332.
16. Wibabara Y, Banura C, Kalyango J, Karamagi C, Kityamuwesi A, et al. (2019) Hepatitis B vaccination status and associated factors among undergraduate students of Makerere University College of Health Sciences. PLOS ONE 14: e0214732.
17. Abdela A, Woldu B, Haile K, Mathewos B, Deressa T (2016) Assessment of knowledge, attitudes and practices toward prevention of hepatitis B virus infection among students of medicine and health sciences in Northwest Ethiopia. BMC Research Notes 9: 410.
18. Bhattarai S, Kc S, Pradhan PMS, Lama S, Rijal S (2014) Hepatitis B vaccination status and Needle-stick and Sharps-related Injuries among medical school students in Nepal: a cross-sectional study. BMC Research Notes 7: 774.
19. Shah DK, Jha RK, Ansari S, Sah P, Dhungana GP, et al. (2016) Knowledge and awareness regarding hepatitis B among preclinical medical and dental students of Chitwan Medical College Nepal: a questionnaire-based study. Int J Med Sci Public Health 5: 2316-2321.
20. Alhowaish MA, Alhowaish JA, Alanazi YH, Alshammari MM, Alshammari MS, et al. (2017) Knowledge, attitudes and practices toward prevention of hepatitis B virus infection among medical students at Northern Border University, Arar, Kingdom of Saudi Arabia. Electron Physician 9: 5388-5394.
21. Al-Hazmi A (2015) Knowledge, attitudes, and practice of medical students regarding occupational risks of hepatitis B virus in college of medicine, aljouf university. Ann Med Health Sci Res 5: 13-19.
22. Rathi A, Kumar V, Majhi J, Jain S, Lal P, et al. (2018) Assessment of knowledge, attitude, and practices toward prevention of hepatitis B infection among medical students in a high-risk setting of a newly established medical institution. J Lab Physicians 10: 374-379.
23. Khan N, Ahmed SM, Khalid MM, Siddiqui SH, Merchant AA (2010) Effect of gender and age on the knowledge, attitude and practice regarding hepatitis B and C and vaccination status of hepatitis B among medical students of Karachi, Pakistan. J Pak Med Assoc 60: 450-455.
24. Ahmad A, Munn Sann L, Abdul Rahman H (2016) Factors associated with knowledge, attitude and practice related to hepatitis B and C among international students of Universiti Putra Malaysia. BMC Public Health 16: 611.
25. Hussein N (2020) The Impact of COVID-19 Pandemic on the Elimination of Viral Hepatitis in Duhok City, Kurdistan Region of Iraq. 20: e104643.
26. Hussein NR, Abdullah AS, Majed HS, Naqid IA, Abdi BA, et al. (2020) Recurrence Rate of Hepatitis C Virus After Achieving a Sustained Virologic Response in Kurdistan Region, Iraq. Avicenna J Clin Microbiol Infect 7: 72-74.
27. Mosa AA, Ibrahim SV, Naqid IA, Hawezy DJ, Al-Jaf SMA, et al. (2023) The Impact of SARS-CoV-2 Pandemic on Medical Students: Knowledge, Attitudes, and Practices towards E-Learning: An Online Cross-Sectional Study in the Kurdistan Region, Iraq. Galician Medical Journal 30: E202314.
28. Daniel S, Mohammed AS, Ibrahim N, Hussein NR, Balatay AA, et al. (2022) Human papillomavirus (HPV) genotype prevalence and impact of COVID-19 on the HPV prevention program in Duhok city. Dialogues Health 1: 100055.
