# Supplementary material for: Hepatitis B vaccination status and knowledge, attitude, and practice towards Hepatitis B virus among medical sciences students: A cross-sectional study
Source: PLoS One. 2023 Nov 6;18(11):e0293822. doi: 10.1371/journal.pone.0293822 (PMC10627443; doi:10.1371/journal.pone.0293822)
Supplement: Attachment — Submitted filename: Response to Reviewers.docx [file pone.0293822.s003.docx]

**The Response to Reviewers Letter**

**PLOS ONE JOURNAL**

**Dear Editor**

Thank you for your kind comments, we found the editorial board and reviewers' comments on our submitted manuscript draft to be very valuable. We believe that our paper is significantly strengthened after responding to the comments.

We have carefully reviewed the comments and have modified the manuscript accordingly. The revised points are highlighted in **RED** in the revised manuscript file.

**Response to “ACADEMIC EDITOR” comments**

1. The manuscript needs extensive proof-reading for English language

- **The manuscript has been revised for typo-grammatical errors by an English language editor**

2. In the abstract, you should provide what was the scale used. Giving just a score of 20 or 26 has no sense

- **Five-point Likert scale has been added to the abstract in the materials and methods section.**

3. Improve the quality of your tables.

- **Done**

4. You should provide the references used to categorize the scores. In fact, considering those who have a score of 64 with those having an eventual score of 10 is ambiguous.

-**Addressed as appropriate in Materials and Methods section**

5. Figure 1 is not necessary.

-**Removed**

6. Table 1 may be "demographics and HBV vaccination status.

**-Done**

7. In table 6 you can include the other factors that could be associated with KAP

**-Done**

All comments provided by the editorial board are adequately responded to.

**Response to “REVIEWERS” Comments**

- **Reviewer #1**

1. The manuscript needs to be revised by a native English speaker as there are many typographical errors and grammatical mistakes.

- **The manuscript has been revised for typo-grammatical errors by an English language editor**

2. Comments on introduction

-**All comments have been addressed**

3. Why is 2010 Global Burden of Disease study being quoted when there are far more recent reports from the GBD.

-**Reference has been updated**

4. Methodology is sound but give information on if the study was piloted and what language the questionnaire was in. Reference 12 (Nepal study) that the study was based on was in English I presume, so if there was a translated questionnaire, was anything done to ensure validity and reliability of the translated questionnaire?

-**Medical sciences colleges in the Kurdistan region of Iraq are in English language. So, all the students are able to answer the questionnaire in English language. Therefore, it did not require translation of the questionnaire.**

5. Discussion focuses too much on comparison with the Nepal study and a Saudi one. Was a thorough literature search conducted to gain more of a worldwide view on the matter.

– **The study was compared to neighboring countries and other developing countries**.

- **Reviewer #2**

-**All the comments provided by reviewer #2 in the reviewed Microsoft Word file are addressed as appropriate.**

**Modification to References**

- Reference number [2] has been updated as appropriate.
- Reference number [12] has been added to last sentence of Material and Methods section/Study tool subheading.

**Note: All data are available within the manuscript and supporting information without any restriction**

We hope that the manuscript is now suitable for your journal, looking forward to hearing from you.

Sincerely,

Asst. Prof. Dr Ibrahim A. Naqid

Department of Biomedical Sciences,

College of Medicine, University of Zakho

Zakho International Road, Duhok, Kurdistan Region-Iraq, P.O. Box12

Email: [ibrahim.naqid@uoz.edu.krd](mailto:ibrahim.naqid@uoz.edu.krd)

Tel:. 009647504737593
