# Supplementary material for: Hepatitis B vaccination status and knowledge, attitude, and practice towards Hepatitis B virus among medical sciences students: A cross-sectional study
Source: PLoS One. 2023 Nov 6;18(11):e0293822. doi: 10.1371/journal.pone.0293822 (PMC10627443; doi:10.1371/journal.pone.0293822)
Supplement: Attachment — Submitted filename: Response to Reviewers R2.docx [file pone.0293822.s005.docx]

**The Response to Reviewers Letter**

**PLOS ONE JOURNAL**

**Dear Editor**

Thank you for your kind comments, we found the remaining unaddressed reviewer’s comments on our submitted manuscript draft to be very valuable. We believe that our paper is further strengthened after responding to the remaining comments.

We have carefully reviewed the comments and have modified the manuscript accordingly. The revised points are highlighted in **BLUE** in the revised manuscript file.

**Response to remaining “REVIEWERS” Comments**

- **Reviewer #2**

1. Materials and Methodology: Year of study of the students?

- **Unfortunately, has not been included in our questionnaire.**

2. Results: Dividing the favorable and non-favorable at median?

-**It is cited in the methodology that we followed reference 12.**

3. Discussion: Reference that supports the below statement “Medical students are at comparable risk of getting hepatitis B compared to Health care workers”

-**A new reference has been added.**

4. Discussion: Comparing to other Pakistan study, reference 23 in the old file and reference 18 in the new file

-**Done.**

5. Discussion: Does Nepali private hospitals offer vaccination services with some cost. Then cost could be one of the barriers for not vaccination.

-**Private hospitals in Nepal provide Hepatitis B vaccination with some cost, but according to what’s available in the literature, we found that cost is not a major barrier to non-vaccination among Nepalese students. For example, in reference 12 only 2 students (1.1%) stated high cost of vaccination as a barrier.**

6. A study has been added to support our recommendations

**Modification to References**

- Two references have been added newly, reference number [13] and [30].
- Reference number [23] has been changed to reference number [18].

We hope that the manuscript is now suitable for your journal, looking forward to hearing from you.

Sincerely,

Asst. Prof. Dr Ibrahim A. Naqid

Department of Biomedical Sciences,

College of Medicine, University of Zakho

Zakho International Road, Duhok, Kurdistan Region-Iraq

P.O. Box12

Email: Ibrahim.naqid@uoz.edu.krd

Tel:. 009647504737593
