# Supplementary material for: Hepatitis B vaccination status and knowledge, attitude, and practice towards Hepatitis B virus among medical sciences students: A cross-sectional study
Source: PLoS One. 2023 Nov 6;18(11):e0293822. doi: 10.1371/journal.pone.0293822 (PMC10627443; doi:10.1371/journal.pone.0293822)
Supplement: Attachment — Submitted filename: Response to Reviewers R3.docx [file pone.0293822.s006.docx]

**The Response to Reviewers Letter**

**PLOS ONE JOURNAL**

**Dear Editor**

Thank you for your kind comments, we found the remaining unaddressed reviewer’s comments on our submitted manuscript draft to be very valuable. We believe that our paper is further strengthened after responding to the remaining comments.

We have carefully reviewed the comments and have modified the manuscript accordingly. The revised points are highlighted in **Green** in the revised manuscript file.

**Response to remaining “REVIEWERS” Comments**

**In all tables No. changed into “n” in green color**

We hope that the manuscript is now suitable for your journal, looking forward to hearing from you.

Sincerely,

Asst. Prof. Dr Ibrahim A. Naqid

Department of Biomedical Sciences,

College of Medicine, University of Zakho

Zakho International Road, Duhok, Kurdistan Region-Iraq

P.O. Box12

Email: Ibrahim.naqid@uoz.edu.krd

Tel:. 009647504737593
